# MiR-487b suppressed inflammation and neuronal apoptosis in spinal cord injury by targeted Ifitm3

**DOI:** 10.1007/s11011-022-01015-3

**Published:** 2022-07-08

**Authors:** Dake Tong, Yanyin Zhao, Yang Tang, Jie Ma, Miao Wang, Bo Li, Zhiwei Wang, Cheng Li

**Affiliations:** 1 Shanghai Key Laboratory of Orthopaedic Implants, Department of Orthopaedic Surgery, Shanghai Ninth People’s Hospital, Shanghai Jiaotong University School of Medicine, 639 Zhizaoju Road, Shanghai, 200011 People’s Republic of China; 2 Department of Neurology, Huashan Hospital, Fudan University, Shanghai, China; 3 Department of Orthopedic Surgery, The Third Affiliated Hospital of Naval Medical University, 700 North Moyu Road, Shanghai, 201805 China; 4 Department of Orthopedics, Changhai Hospital, Naval Medical University, 168 Changhai Road, Shanghai, 200433 People’s Republic of China

**Keywords:** Spinal cord injury (SCI), Inflammation, Bioinformatics, ceRNA network, MiR-487b, Ifitm3

## Abstract

Spinal cord injury (SCI) was a serious nerve injury, which involves complex genetic changes. This paper was intended to investigate the function and mechanism of differentially expressed genes in SCI. The three datasets GSE92657, GSE93561 and GSE189070 of SCI from GEO database were used to identify differentially expressed genes (DEGs). We identified the common DEGs in the three datasets GSE92657, GSE93561 and GSE189070 of SCI from GEO database. Next, a protein-protein interaction (PPI) network of DEGs was constructed. Subsequently, the Gene Ontology (GO) and Kyoto Encyclopedia of Genes and Genomes (KEGG) analysis showed that DEGs were significantly enriched in immune response, inflammatory response. The expression level of immune-related genes (Arg1, Ccl12, Ccl2, Ifitm2, Ifitm3, and et al.) at different time points of SCI were analyzed in GSE189070 dataset. Next, differentially expressed miRNAs (DE-miRNAs) were identified in SCI compared with normal based on GSE158194 database. DE-miRNA and targeted immune-related genes were predicted by miRwalk, including miR-487b-5p targeted Ifitm3, miR-3072-5p targeted Ccl3, and et al. What’s more, the miR-487b was identified and verified to be down-regulated in Lipopolysaccharide (LPS)-induced BV-2 cell model. Further, the miR-487b inhibited cell inflammation and apoptosis in LPS-induced BV2 cell by targeted Ifitm3. For the first time, our results revealed that miR-487b may play an important regulatory role in SCI by targeted Ifitm3 and provide further evidence for SCI research.

## Introduction

Spinal cord injury (SCI), continues to be a severe health problem worldwide, usually leads to permanent motor and sensory disturbances, which seriously affects the quality life of the people. The primary reasons of leading to SCI were mechanical injury (Ahuja et al. 2017), iatrogenic surgery (Hewson et al. 2018), tumor(Ge et al. 2019) or infection (Krause et al. 2016). There was a series of complications after SCI such as neuropathic pain (Zhang and Yang 2017), cardiovascular dysfunction (West et al. 2013), gastrointestinal dysfunction (Holmes and Blanke 2019), and cancer (Nahm et al. 2015). Because of the molecular mechanisms and pathophysiological events of SCI are complicated, which also makes it difficult to cure SCI (Fakhoury 2015).

According to previous work and the progress of SCI research, inflammatory reaction play a pivotal role in the progression of SCI, which contributes to secondary tissue damage that leads to further functional loss (David et al. 2012). Studies have reported that chemokines, immune cytokines, and apoptosis factors are differentially expressed after SCI. Moreover, immune response plays an important role in many diseases. For example, immune cell infiltration of M1 and M2 macrophages, natural killer, NKT cells, effector and memory T cells, and granulocytes was a prominent feature in dysfunctional adipose tissue (Guzik et al. 2017), A sustained active immune response usually leaded to chronic inflammation, which was characterized by prolonged acuteness over time and simultaneous tissue destruction and repair. The infiltration of immune cells in the myocardium adversely affected the heart and led to the pathogenesis of heart failure (Carrillo-Salinas et al. 2019). Therefore, to uncover the change of pivotal molecules that participate in the immune/inflammation response of SCI is important.

Non-coding RNAs (ncRNAs) have been found play a very important role in the pathogenesis of SCI (Guo et al. 2019; Viereck and Thum 2017;Wang et al. 2019), which includes microRNA (miRNA). Increasingly studies have shown that miRNA is involved in the secondary injury and repair process after SCI. After SCI, dysregulated miRNAs can participate in inflammatory responses, as well as the inhibition of apoptosis and axon regeneration through multiple pathways(Liu et al. 2020). However, the functions of miRNAs in SCI progression need further specific elucidation.In this study, we aim to investigate the role and functions of differentially expressed genes (DEGs) in SCI by bioinformatics analysis. We next explore the function of miR-487b and its targeted gene interferon-induced transmembrane protein 3 (Ifitm3) in LPS induced BV2 microglial, it have a strong correlation with immune/inflammation response. These findings provide further evidence for SCI research.

## Materials and methods

### Data acquisition

The microarrays data of mRNAs expression profile of SCI and normal tissues obtained from Gene Expression Omnibus (GEO) database (http://www.ncbi.nlm.nih.gov/geo), which contained three datasets GSE92657 (Lou et al. 2017), GSE93561 (Takano et al. 2017) and GSE189070. All datasets were based on the SCI models of C57BL/6J mice to analysis the microarrays data of mRNA expression profile. GSE92657 included 3 SCI and 3 normal samples, GSE93561 included 6 SCI and 6 normal samples. GSE189070 included transcriptomic profile of astrocytes from uninjured spinal cord tissue and nearby the lesion epicenter at 3, 7, 14 days after mouse hemisection spinal cord tissue.

### Identification of DEGs

We carried out GEO2R which is an interactive web tool that could be used to identify DEGs. After the RNA-seq data of SCI and the sham operation groups normalized, the differential expression of mRNA (DE-mRNAs) or miRNAs (DE-miRNAs) were analyzed using the R package DEseq2, which with the threshold of adjusted P-value < 0.05 and fold change ≥ 2. We used the miRwalk website (http://mirwalk.umm.uni-heidelberg.de/) to predict miRNA-mRNA interaction information.

### PPI network construction

The overlap DE-mRNAs of the GSE92657 and GSE93561 datasets obtained by Venn 2.1.0 (https://bioinfogp.cnb.csic.es/tools/venny/) analysis. To evaluate the interacting relationship of overlapped genes, we analyzed these genes using the Search Tool for the Retrieval of Interacting Genes/Proteins (STRING) online database (version 11.0, http://string-db.org/). The minimum required interaction score is default as 0.4. Next, a visualized PPI network of these DE-mRNAs was constructed using Cytoscape software (version v3.7.2, https://cytoscape.org/) (Doncheva et al. 2019; Saha et al. 2020; Shannon et al. 2003). The plug-in MCODE of Cytoscape was used to identify the most significant module of the PPI network.

### Function enrichment analysis

The Gene Ontology (GO) annotations and Kyoto Encyclopedia of Genes and Genomes (KEGG) pathway analysis were applied to investigate the roles of all DE-mRNAs. GO annotations including biological process (BP) was performed using DAVID database (https://david.ncifcrf.gov) (Dennis et al. 2003) KEGG network was constructed by Cytoscape ClueGo. The pathways were significant enrichment with *P*-value < 0.05.

### Cell line and cell culture

Microglial BV2 cells were obtained from the Institute of Basic Medical Sciences of the Chinese Academy of Medical Sciences (Beijing, China). BV2 microglial cells were cultured in DMEM containing 10% fetal bovine serum, penicillin 100 U/mL, and streptomycin 100 µg/mL at 37 °C in a humidified atmosphere of 5% CO_2_. Cells were cultured in serum-free DMEM for at least 4 h before treatments. The LPS-induced BV2 cells were treated with 1 µg/mL LPS, and the control group treated with the same volume of culture medium. After 20 h, the culture medium supernatant and cells were collected.

### Cell transfection

Plasmids were purchased from Sangon Biotech (Shanghai). BV2 microglia were transfected with the miRNA-487b mimics plasmid using Lipofectamine 2000 (Invitrogen, Rockville, MD, USA) according to the manufacturer’s instructions, while the control group was transfected with the empty plasmid. After 6 h, the cells were washed and maintained in culture for 48 h for further analysis. The transfection efficiency was determined by detecting fluorescence.

### ELISAs for inflammatory factors

Mouse factors tumor necrosis factor (TNF)-α and interleukin (IL)-6 in ELISA kits (Proteintech, Wuhan, China) were used to detect cytokine concentrations in supernatants of microglial cultures. Briefly, 100 µL of cultured media from different groups were added to each well of 96-well plates coated with anti-mouse cytokine antibodies. The plates were incubated at 37 °C for 90 min and then washed 5 times. Next, 100 µL of biotinylated cytokine-specific antibody were added to each well and incubated at 37 °C for 60 min. Then, the plates were washed, treated with 100 µL of diluted streptavidin-HRP, and incubated at 37 °C for 30 min. The color was produced by the addition of 100 µL of substrate solution and an incubation for 10–15 min after washing. Finally, 100 µL of stop solution were added to terminate the reaction. Finally, the optical density at 450 nm was measured within 10 min.

### qRT-PCR

Total RNA was extracted from BV2 cells using a commercial TRIzol kit (Invitrogen, USA), and then RNA was reverse-transcribed into cDNAs with a PrimeScript RT reagent Kit (Takara, Dalian, China). The quantitative experiment was completed using an ABI 7500 PCR instrument (Applied Biosystems, USA) and a SYBR green Kit (Applied Biosystems, USA), with the relative gene expression levels normalized to GAPDH. Primers are shown in Table 1.

**Table 1:** Specific RNAs primers for quantitative qRT-PCR analysis

Gene name	Sequence
GAPDH	F: GCCAAGGCTGTGGGCAAGGT
	R: TCTCCAGGCGGCACGTCAGA
U6	F: ATTGGAACGATACAGAGAAGATT
	R: GGAACGCTTCACGAATTTG
miR-709	F:GGGGGAGGCAGAGGCA
	R:CAGTGCGTGTCGTGGAGT
miR-149-5p	F:GGGTCTGGCTCCGTGTCTC
	R:CAGTGCGTGTCGTGGAGT
miR-3071	F:GGGACTCATGAGACGAT
	CAGTGCGTGTCGTGGAGT
miR-1941-5p	F:GGGAGGGAGATGCTGGTACA
	R:CAGTGCGTGTCGTGGAGT
miR-6963-3p	F:GGGTGCCTCTGCCTCCATC
	R:CAGTGCGTGTCGTGGAGT
miR-487b-5p	F:GGGTGGTATCCCTGTC
	R:CAGTGCGTGTCGTGGAGT
miR-12191-3p	F:GGGCCCATGGAGCTGTAG
	R:CAGTGCGTGTCGTGGAGT
miR-21a-3p	F:GGGCAACAGCAGTCGATGG
	R:CAGTGCGTGTCGTGGAGT
Ifitm3	F: GAGGACAGCCCCCAAACTAC
	R: CTCCAGTCACATCACCCACC

### Cell apoptosis assay

For the assessment of cell death levels, cultured BV2 cells were collected after transfection and rinsed with chilled PBS, followed by an incubation with Annexin V-fluorescein isothiocyanate (FITC)/propidium iodide(PI) (No.C1062S, Beyotime, Nanjing, China) staining in the dark for 15 min. Then, the percentages of apoptotic cells were detected using flow cytometry (Beckman Coulter, Brea, CA, USA).

### Dual-luciferase reporter assay

The miR-487b sequence in the BV2 cells was subcloned into the luciferase reporter psiCHECK2 (Promega, Madison, WI, USA) and designated as psiCHECK2- circ-Usp10-WT. The circ-Usp10 sequence with mutation of miR-152 binding site was synthesized using overlap extension PCR and cloned into psiCHECK2 vector designated as psiCHECK2- circ-Usp10-Mut. The mutant vector for the miR-152 binding site was constructed and termed as psiCHECK2-miR-152-3′UTR-Mut. A total of 3 × 10^4^ BV2 cells were seeded in 24-well plates in triplicate. At 48 h following transfection with miR-152 mimics, luciferase reporter assays were conducted using the dual-luciferase reporter assay system (Promega) according to the manufacturer’s instructions. Relative luciferase activity was normalized to the Renilla luciferase internal control.

### Statistical analysis

We performed correlation analysis using Student’s t test by GraphPad Prism 8, *p*-value < 0.05 was considered statistically significant. Data processing and analysis using Microsoft Excel and R software (R software, version 3.5.1).

## Results

### Identification of the common DEGs in SCI tissue

**Fig. 1:**
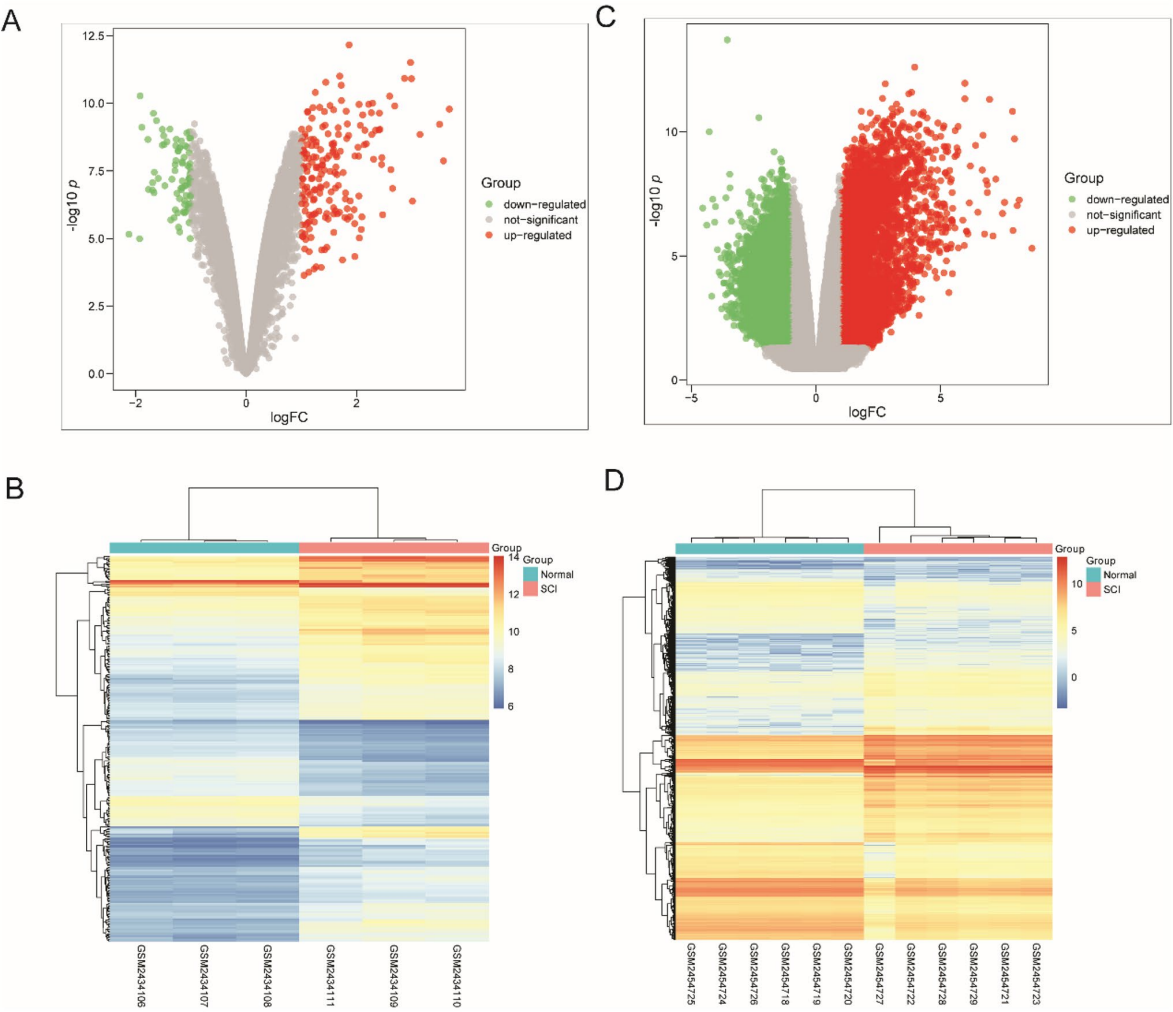
The differentially expressed (DE) mRNAs in SCI tissue. (**A**-**B**) Volcano plot (**A**) and heat map (**B**) of DE-mRNAs in the SCI group compared with the control group in datasets GSE92657. (**C**-**D**) Volcano plot (**C**) and heat map (**D**) of DE-mRNAs in the SCI group compared with the control group and in GSE93561. Up-regulated and down-regulated genes were colored with red and green, respectively

**Fig. 2:**
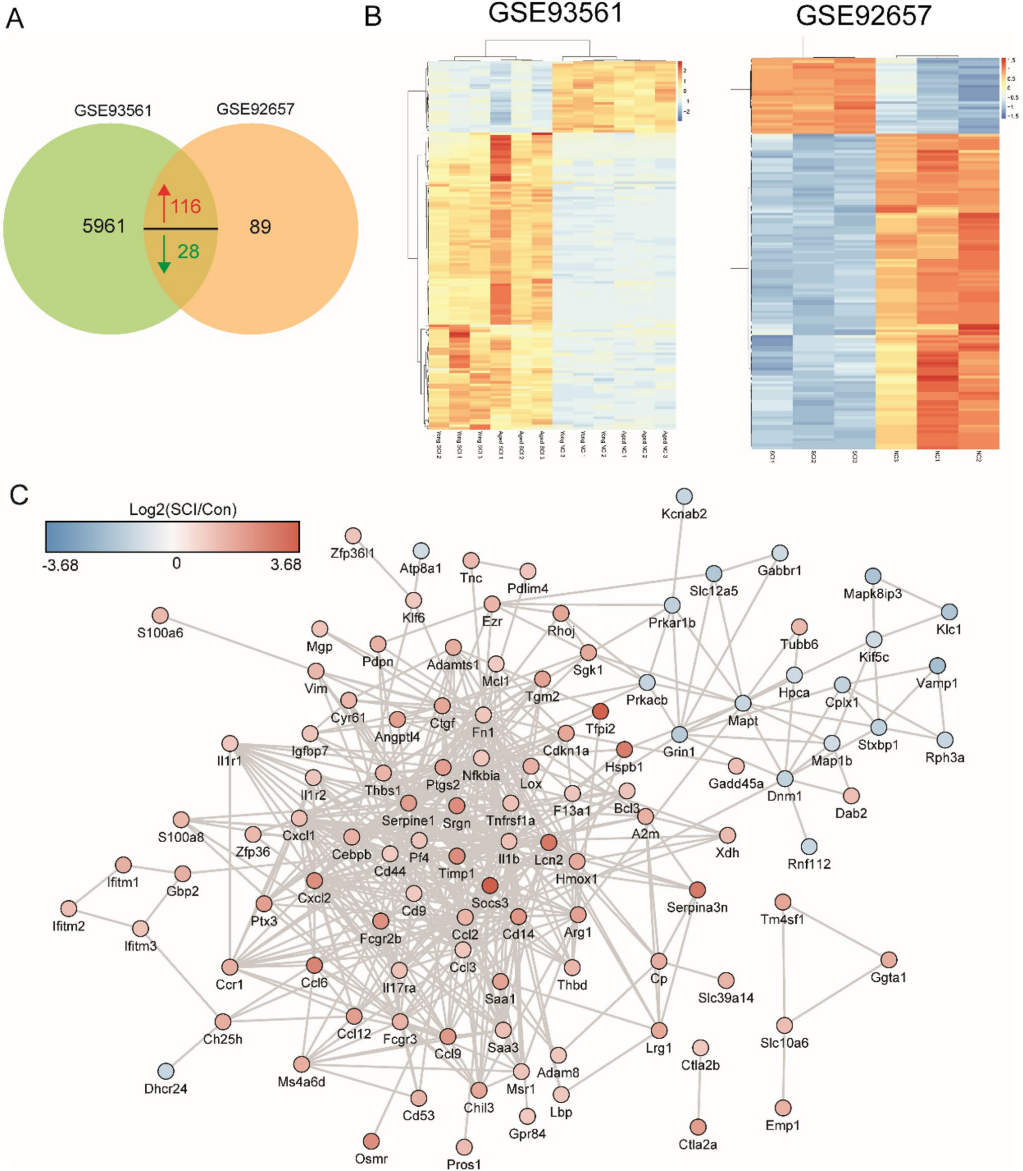
Identification of the common DE-mRNAs. Venn diagram showing the overlap of the DE-mRNAs between the datasets of GSE92657 and GSE93561. The heatmap showing the relative expression of common DE-mRNAs in datasets GSE92657 and in GSE93561. The PPI network of common DE-mRNAs was constructed using Cytoscape software. The red represents up-regulation genes, blue represents down-regulation

To explore the difference in molecular expression between SCI tissue and normal spinal cord tissue, DE-mRNAs were screened out by microarray data analysis. P-value < 0.05 and fold change ≥ 2 were used as the threshold of screening differentially expressed genes. As shown in Fig. 1A and C  of the volcano plot, a total of 233 and 6105 DE-mRNAs were obtained from the datasets GSE92657 and GSE93561, respectively. Otherwise, to have a clearer understanding of the expression distribution of differential genes in the SCI group and the normal group, we perform heatmap cluster analysis on DE-mRNAs (Fig. 1B and D). Subsequently, we performed Venn analysis to obtain more credible DE-mRNAs. As shown in Fig. 2A, there were 144 common DE-mRNAs in the datasets GSE92657 and GSE93561, of which 116 were up-regulated genes and 28 were down-regulated genes. The heatmap showing the relative expression of common DE-mRNAs in datasets GSE92657 and in GSE93561 (Fig. 2B). For intuitively understand the interaction of these DEGs, we constructed a PPI network showing the high expression gene network and low expression network (Fig. 2C).

### Identification of the immune-related genes and their timely change in SCI

**Fig. 3:**
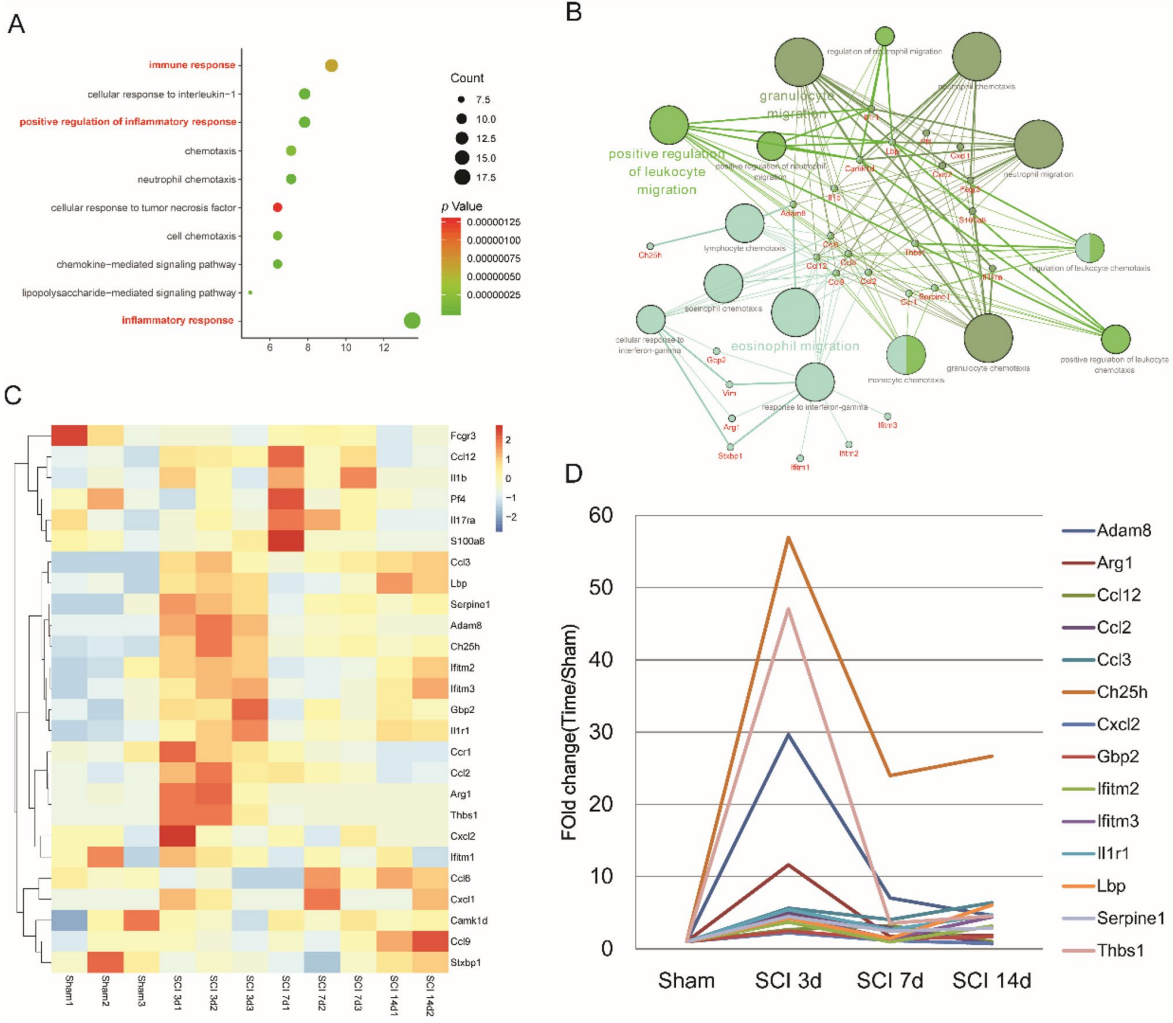
Identification of the immune-related genes and their timely change in SCI. The top 10 enriched biological process (BP) of the common DE-mRNAs. The representative biological processes of signaling pathways for the DEGs. Targeted genes were predicted and functional pathway was established; The important terms in the group were tagged, with the related biological functional groups partially; The expression level of immune-related genes at different time points of SCI in GSE189070 dataset. The relative expression level of immune-related genes at different time points of SCI (3-day (3d), 7d, 14d) in GSE189070 dataset

To further explore the function of common DE-mRNAs shared by the GSE92657 and GSE93561 in the biological processes (BP) afterSCI, we performed GO using DAVID. The top 10 significantly enriched terms of BP were shown in Fig. 3A. The terms of immune response, positive regulation of inflammatory response, and inflammatory response were significantly enriched, indicating that the immune response plays an important role in the progress of SCI, which is consistent with a previously reported study (Alizadeh et al. 2018). Besides, the representative BP of signaling pathways for the DEGs was constructed based on STRING database and Cytoscape (ClueGO) (Fig. 3B). It is found that positive regulation of leukocyte migration, neutrophil chemotaxis, neutrophil chemotaxis and so on signaling pathways showed consistent higher correlation with DEGs. Genes like Cxcl2, II1b, and Ccl2 contribute to the regulation signaling pathways. The above results indicated that SCI is associated with inflammation and immune response. What’s more, to further verify the timely change of immune-related genes, we also verify their expression level in the testing dataset GSE189070 (Fig. 3C). As demonstrated in Fig. 3D, most of the immune-related genes like Ch25h, Thbs1, Cxcl2, Gbp2 and Ccl3, etc. were up-regulated expressed in 3th day, indicating that these genes were activated at this time point.

### Identification and validation of the DE-miRNAs in SCI

**Fig. 4:**
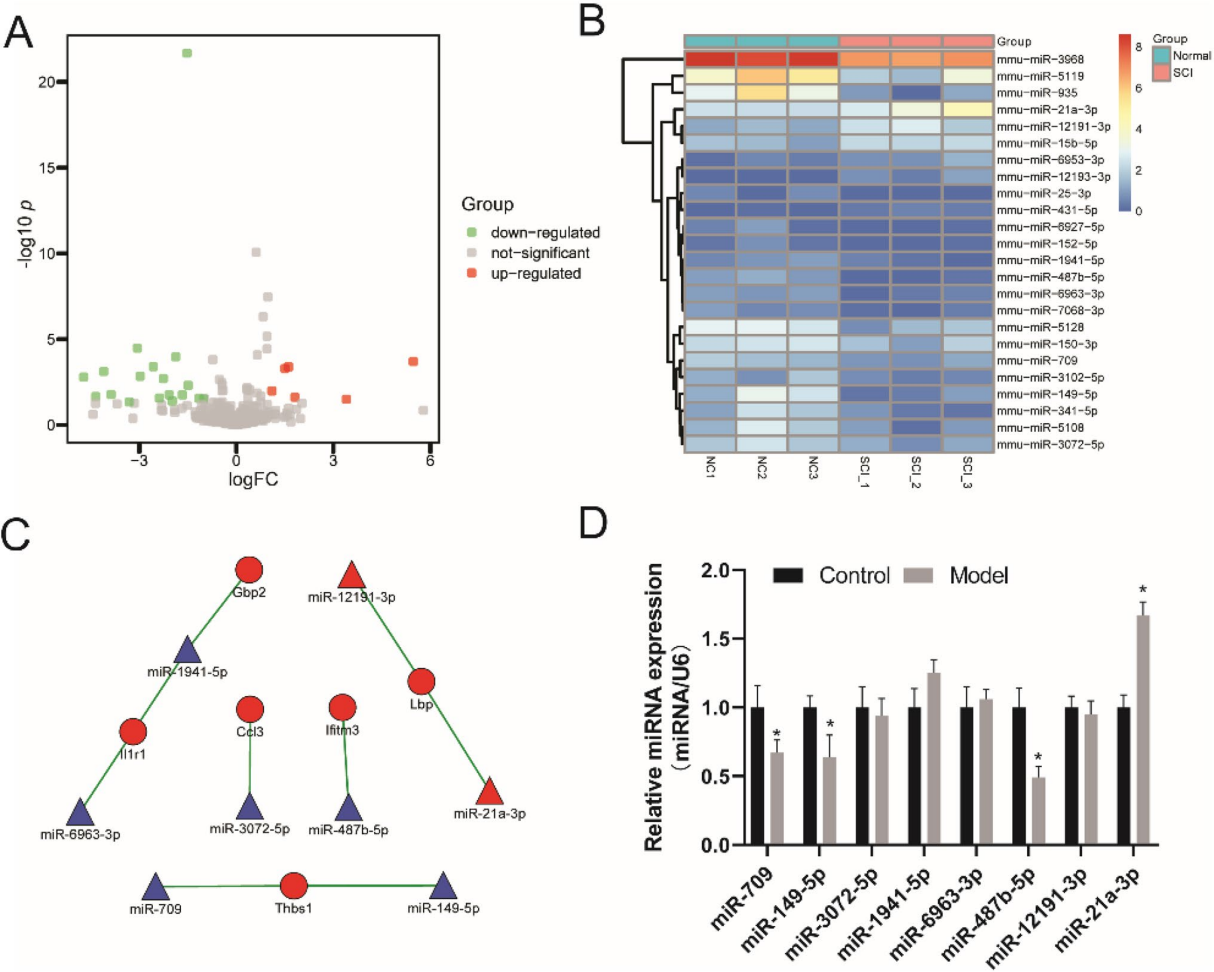
Identification and validation of DE-miRNAs. (**A**-**B**) Volcano plot (**A**) and heat map (**B**) of DE-miRNAs in the SCI group compared with the control group based on GSE158194 database (**C**) The correlation of DE-miRNA and related targeted immune-related genes predicted by mirWalk. Circle represents mRNAs, triangle represents miRNAs, red represents up-regulation and blue represents down-regulation. The relative DE-miRNAs expression in the LPS-induced BV-2 cell compared with the control group. Results are expressed as the Mean ± SEM. *p < 0.05 compared with control group

In the next step, we screened out 24 DE-miRNAs based on dataset GSE158194 as shown in Fig. 4A. Otherwise, to have a clearer understanding of the expression distribution of differential genes in the SCI group and the normal group, heatmap cluster analysis on DE-miRNAs (Fig. 4B) were carried out. Using the immune-related DE-mRNAs identified in Fig. 3, the targeted genes of the DE-miRNAs were predicted by miRWalk, which included 6 up-regulated DE-mRNAs, 6 up-regulated DE-miRNAs and 2 down-regulated miRNAs (Fig. 4C). To further verify the expression level of these DE-miRNA in the SCI, we carried out the model of SCI in vitro (BV2 cell line) induced by LPS. The relative expression level of miRNAs was shown in Fig. 4D, which miR-709, miR-149-5p and miR-487b-5p were significantly down-regulated and miR-21a-3p was significantly up -regulated. Because the expression level of miR-487b-5p was down-regulated most significantly among the three miRNAs, we choose miR-487b-5p for the following further investigation.

### MiR-487b reduced LPS-induced BV2 cell inflammation and apoptosis by targeted Ifitm3

**Fig. 5:**
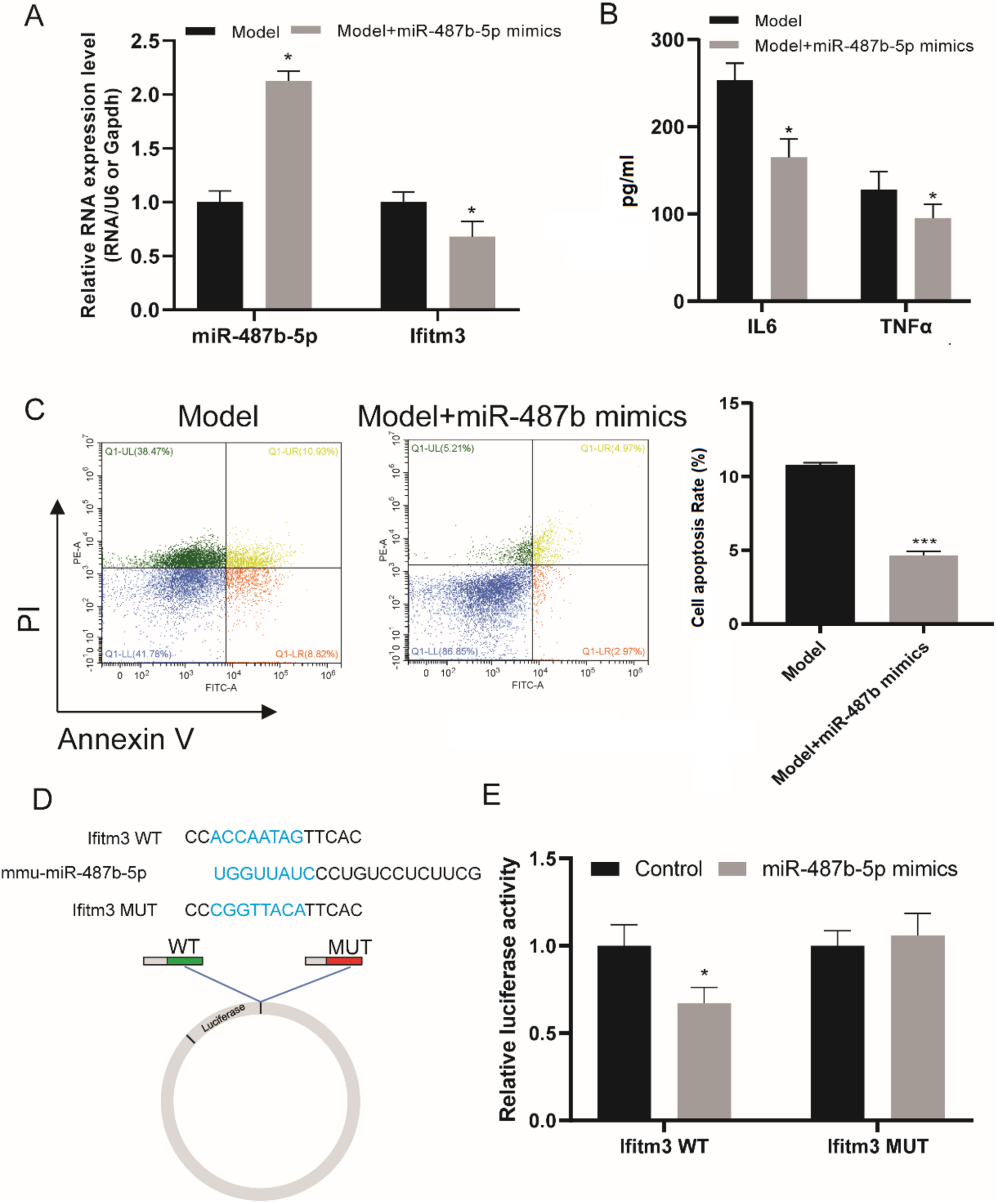
MiR-487b reduced LPS-induced BV2 cell inflammation and apoptosis by targeted Ifitm3. The relative RNA expression level of miR-487b and Ifitm3 in SCI model group and SCI + miR-487b mimics group. The expression of IL9 and TNFα (pg/mL) in SCI model group and SCI + miR-487b mimics group. Representative flow cytometry showing that the miR-487b mimics inhibits cell apoptosis in SCI model. ( FITC-A: fluorescein isothiocyanate signal area; PE-A: Phycoerythrin signal area). The binding part sequencing of mmu-miR-487b-5p, Ifitm3 WT and Ifitm3 MUT used for luciferase reporter assay. Luciferase reporter assay was performed in miR-487b mimics and control group. Results are expressed as the Mean ± SEM. *p < 0.05, ***p < 0.001 compared with control group

SCI result in microglial and astrocyte activation, neuroinflammation and neuronal cell death (Hausmann 2003). Microglia is the reactive resident of neuroinflammation at the injury site (Plemel et al. 2014). After SCI, microglia can play important role (Bowes and Yip 2014). We next explore the function of miR-487b and its targeted gene interferon-induced transmembrane protein 3 (Ifitm3) in LPS induced BV2 microglial. As shown in Fig. 5A, when the BV2 cell treated with LPS and miR-487b mimics, the relative expression of miR-487b was significantly enhanced compared to control group and decreased the expression level of Ifitm3. Thereafter, we found that miR-487b mimics significantly inhibited the expression of the proinflammatory factors TNF-α, IL-6 in microglia treated with LPS (Fig. 5B). The flow cytometry showing that the miR-487b mimics remarkably inhibitscell apoptosis in LPS induced BV-2 (Fig. 5C). Luciferase reporter gene experiments verified the tight binding of miR-487b and Ifitm3 (Fig. 5D and E). The results above revealed that miR-487b reduced LPS-induced BV2 cell inflammatory response and apoptosis by targeted Ifitm3.

## Discussion

According to previous reports, most spinal cord injuries were caused by mechanical injuries, and there were also some non-mechanical factors, including degenerative CNS disorders, genetic and metabolic, infectious, inflammatory, ischemia, and other pathogenic factors (El Masri and Kumar 2011; Lynch et al. 2016; McDonald and Sadowsky 2002; van Middendorp et al. 2011). Although the pathogenic factors were well known, there are still great challenges in the rehabilitation after SCI, mainly due to the nerve injury and complexity of molecular mechanism after SCI (Tran et al. 2018; van Niekerk et al. 2016). In addition, the immune/ inflammation related genes of mice tissues of SCI were worth more attention. Here, our research was mainly devoted to explore the key genes in SCI, and to identify the immune-related differentially expressed genes.

First, we selected two datasets GSE92657 and GSE93561 from public database GEO, which contained SCI and normal spinal cord tissue RNA-seq data. In total, 144 common DE-mRNAs in datasets GSE92657 and GSE93561 were obtained by Venn analysis. Subsequently, we performed GO functional enrichment analysis on the differential genes shared by the two datasets. We found that many DEGs were enriched in the biological pathways of immune response and inflammatory response. Hence, we speculate that these DEGs may affect the level of immunity and inflammation of SCI tissues to some extent. We also found that many chemokines were enriched in the PPI network, and the genes enriched were related to the occurrence of cell metastasis and inflammation. For instance, FN1 is a member of the fibronectins (FN) family, and once it is overexpressed, the TGF-β/PI3K/Akt signaling pathway can be activated to promote fracture healing (Zhang et al. 2021). Besides, another important molecule of tissue inhibitor of metalloproteinase 1 (TIMP1) play an important role in the SCI, which was consistent with the results of a previous study (Liu et al. 2015). Therefore, we speculated that these changes at the molecular level of DEGs greatly regulate the immunity and inflammation level of SCI.

MicroRNAs play a significant role in the regulation of SCI. After SCI, dysregulated miRNAs can participate in inflammatory responses, as well as the inhibition of apoptosis and axon regeneration through multiple pathways(Liu et al. 2020). Sun et al. indicated that miRNA-411 attenuates inflammatory damage and apoptosis following SCI(Sun et al. 2020). Chen et al. found that miRNA-194-5p inhibits inflammatory response after SCI via regulating TRAF6(Chen et al. 2020). Zhang et al. suggest that miR-223 targets NLRP3 to relieve inflammation and alleviate SCI. In the present study, we identified the miR-487b and found that the expression level of miR-487b was down-regulated significantly, which is consistent with the previous study that miR-487b was observed to target cholesterol metabolism-associated DGEs in rats with SCI (Chen et al. 2015). Besides, overexpression of miR-487b in BV-2 cell was observed to alleviative the proinflammatory factors, indicating its regulation role in inflammatory responses in SCI. What’s more, the results of luciferase reporter gene experiments verified the tight binding of miR-487b and Ifitm3. Ifitm3 was identified as an innate immunity protein that predominantly associated with Alzheimer’s disease(Hur et al. 2020) and cancers(Rajapaksa et al. 2020). For the first time, we suspect that Ifitm3 is an important molecule that has changed after SCI and miR-487b reduced LPS-induced BV2 neuronal apoptosis by targeted Ifitm3. These results suggest that the aberrant miR-487b is possibly regulated immune/inflammation signaling pathway and continuously affects the physiological and biochemical status of cells, thus participating in the regulation of SCI.

## Conclusions

In conclusion, we identified common DEGs based on the public datasets and found that these DEGs were predominantly associated with immune and inflammation response by functional enrichment analysis. Further, the miR-487b was identified and verified to down-regulated in SCI. The miR-487b suppressed inflammation and reduced LPS-induced BV2 neuronal apoptosis by targeted Ifitm3. For the first time, our results reveal that miR-487b may play an important regulatory role in SCI by targeted Ifitm3 and provide further evidence for SCI research. The specific molecular regulation mechanism needs to be embodied in the further experiments.

## Data Availability

The datasets used and analyzed during the current study are available from the corresponding author on reasonable request. The data used in this study can be found in GEO database, including GSE92657, GSE93561 and GSE189070 of SCI.
